# Single-Cell Transcriptomics Supports a Role of *CHD8* in Autism

**DOI:** 10.3390/ijms22063261

**Published:** 2021-03-23

**Authors:** Anke Hoffmann, Dietmar Spengler

**Affiliations:** Translational Research in Psychiatry, Max Planck Institute of Psychiatry, 80804 Munich, Germany; hoffmann@psych.mpg.de

**Keywords:** ASD, CHD8, single-cell sequencing, excitatory/inhibitory imbalance, neocorticogenesis, CRISPR/Cas9 editing, cerebral organoids, neocorticogenesis

## Abstract

Chromodomain helicase domain 8 (*CHD8*) is one of the most frequently mutated and most penetrant genes in the autism spectrum disorder (ASD). Individuals with *CHD8* mutations show leading symptoms of autism, macrocephaly, and facial dysmorphisms. The molecular and cellular mechanisms underpinning the early onset and development of these symptoms are still poorly understood and prevent timely and more efficient therapies of patients. Progress in this area will require an understanding of “when, why and how cells deviate from their normal trajectories”. High-throughput single-cell RNA sequencing (sc-RNAseq) directly quantifies information-bearing RNA molecules that enact each cell’s biological identity. Here, we discuss recent insights from sc-RNAseq of CRISPR/Cas9-editing of *Chd8/CHD8* during mouse neocorticogenesis and human cerebral organoids. Given that the deregulation of the balance between excitation and inhibition (E/I balance) in cortical and subcortical circuits is thought to represent a major etiopathogenetic mechanism in ASD, we focus on the question of whether, and to what degree, results from current sc-RNAseq studies support this hypothesis. Beyond that, we discuss the pros and cons of these approaches and further steps to be taken to harvest the full potential of these transformative techniques.

## 1. Introduction

Autism spectrum disorder (ASD) encompasses a spectrum of early-onset neurodevelopmental disorders with an estimated prevalence of ~1.5% in developed countries [1]. Patients present early deficits in social interaction and communication, repetitive patterns of behavior, and restricted interests and activities [2]. ASD occurs more frequently in boys than in girls and associates with various co-morbidities, such as intellectual disability (ID) (35%), language delay (50%) or epilepsy (5–15%) [2]. All of these impairments are lifelong and greatly reduce the quality of life with no cure available yet.

Heritability for ASD in monozygotic twins is high (0.62–0.94), and siblings of affected individuals show a high risk for relative recurrence (10.1%) (reviewed in [3]). The underlying genetic architecture is complex and involves many independent loci containing common and rare variants with common variants thought to account for a major part of ASD liability [4]. In 2019, Grove et al. [5] detected in a genome-wide association meta-analysis (18,381 individuals with ASD and 27,969 controls) five common genome-wide significant loci specific to ASD. An additional seven loci were shared with other traits (schizophrenia, major depression and educational attainment) at equally strict significance levels. The identified ASD-candidates showed the highest expression during fetal corticogenesis, thus pointing to a sensitive developmental time window during which genetic risk might play out.

**Figure 1:**
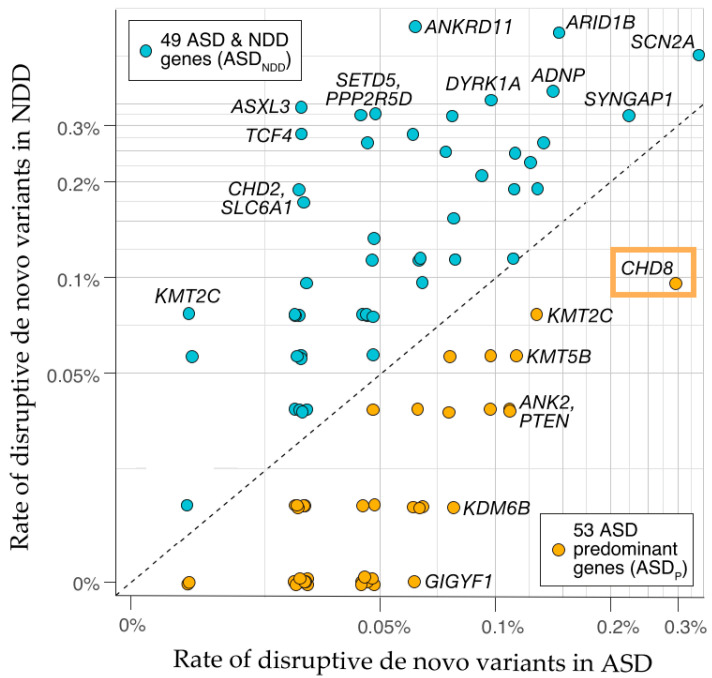
The frequency of disruptive de novo variants, including protein truncation variants and missense variants with an MPC (missense badness, PolyPhen-2, constraint) score ≥1 in autism spectrum disorder (ASD)-ascertained and neurodevelopmental delay (NDD)-ascertained cohorts is shown for the 102-associated genes. Fifty-three genes (orange circles) with a higher frequency in ASD are designated ASD-predominant (ASD_P_), while 49 genes with a higher frequency in NDD (light blue circles) are designated NDD-predominant (NDD_P_). The high confidence risk gene *CHD8* (chromodomain helicase domain 8), boxed in orange, top-ranks among ASD-predominant genes. Graphic adapted from [8], attribution license 5004260244019.

Contrary to inherited variations, de novo mutations, mostly copy number variants (CNVs), and gene disrupting point mutations show larger individual effects but explain in aggregate <5% of the overall liability for ASD [4,6,7]. Just recently, Satterstrom et al. [8] identified in a large-scale exome sequencing study (11,986 individuals with ASD and 23,598 controls) 102 risk genes (FDR ≤ 0.1) that provide important insight into the underpinning of ASD both individually [9,10] and in aggregate [6,11]. Interestingly, 49 of these candidates showed higher frequencies of disruptive de novo variants in individuals diagnosed with severe neurodevelopmental delay (NDD), while 53 showed higher frequencies in individuals diagnosed with ASD. Notably, *CHD8* (chromodomain helicase domain 8) was top-ranked among the 53 predominant ASD genes (Figure 1).

The successful identification of inherited and de novo high confidence risk genes for ASD has nourished the expectation that these genes could serve as entry points to disclose the molecular and cellular foundation of ASD at the level of individual cells and tissues. Against this backdrop, we will scrutinize to what degree recent findings on single-cell transcriptomics of the high confidence risk gene *CHD8* have fulfilled this expectation. Thereby, we will focus on the role of *CHD8* for the development and function of the neuronal excitatory/inhibitory system. Readers more broadly interested in the role of *CHD8* for transcription and animal models are referred to recent reviews [12,13,14]. Initially, we will recapitulate key findings of CHD8 in terms of its gene-regulatory role and as a genetic cause of a distinct neurodevelopmental syndrome with the leading symptoms of autism, macrocephaly, and facial dysmorphisms. Then, we scrutinize clinical and preclinical evidence for the excitatory/inhibitory imbalance hypothesis in ASD. On that basis, we analyze recent results from single-cell transcriptomics of *CHD8* during mouse neocorticogenesis and in human cerebral organoids. The pros and cons of these experimental approaches and further steps to be taken will be considered.

The literature selection process for this review was conducted in the databank PubMed via combinations of the search terms “CHD8”, “autism”, “single-cell sequencing” and “excitation inhibition balance” with date limits from January 2011 to January 2021. Additional searches included scrutiny of similar articles suggested by PubMed, of references from the identified publications, and of citatory publications identified by Google Scholar^®^.

## 2. A Role of *CHD8* in Gene Regulation

The linear DNA-strand of eukaryotic cells is coiled around two copies each of the core histone proteins H2A, H2B, H3 and H4 to form a nucleosome, the basic unit of the chromatin. Further coiling of the chromatinized DNA-strand in 3D-space leads to the formation of a closed, transcriptionally inactive, chromatin structure. Epigenetic processes, such as posttranslational modifications of free histone tails, chromatin remodeling, and DNA modifications, act together to regulate chromatin conformation in a spatially and temporally controlled manner during development and beyond (reviewed in [15,16]. Consistent with this critical function, alterations in epigenetic factors driving these processes have been increasingly recognized as the genetic cause for various neurodevelopmental syndromes (reviewed in [17,18]). For instance, the chromatin remodeler (CR) *CHD8* has been identified as the genetic cause of a distinct neurodevelopmental syndrome (see the following section).

**Figure 2:**
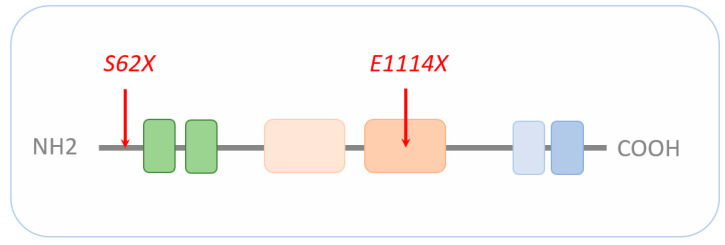
Schematic representation of *CHD8*. The signature motif of the entire CHD family is an N-terminal tandem chromodomain (green boxes) responsible for chromatin binding. The central SNF2-family ATPase domain consists of two lobes (light beige and beige box), with each containing two tandem RecA-like folds parts known as DExx and HELIC. The ATPase domain uses ATP hydrolysis to guide toward translocation down the DNA minor groove. The C-terminus contains functional motifs, such as SANT (light blue) or BRK (blue) domains. SANT domains support association with histone tails, while the BRK domain is also found in several SWI/SNF complexes. The localization of the *CHD8* loss-of-function mutations *S62X* and *E1114X* (see Section 5.5) are highlighted by red arrows. Drawing is not to scale and refers to the long form of CHD8. Schematic adapted from [21], attribution CC BY.

CRs regulate nucleosome sliding, conformational changes of nucleosomal DNA, and exchange of histone variants. All these processes affect the access of transcription factors (TFs) to their binding sites, and consequently, gene expression (reviewed in [19]). CHD8 belongs to a subfamily of CRs that utilize ATP (adenosine triphosphate) hydrolysis to promote translocation down the DNA minor groove (reviewed in [20]). Additional domains next to the central ATPase domain are important to chromatin binding, interaction with specific histone modifications, and/or regulation of ATPase activity. In this regard, CHD8 contains an N-terminal tandem chromodomain mediating binding to methylated lysine residues in free histone tails and a C-terminal SANT-like domain supporting an association with histone tails. In addition, the C-terminus harbors a BRK domain also found in related CHD subfamilies (Figure 2).

This modular structure and combinatorial use of regulatory domains within the CHD family suggests common and subtype-specific roles in chromatin remodeling (discussed in [22]). In general, CHDs, including CHD8, recognize chromatin signatures that can undergo dynamic changes, thus modulating their regulatory space. Such plasticity makes it more difficult to define genuine CHD target genes when compared to genes targeted by DNA sequence-specific TFs [12,14].

## 3. Disruptive de Novo Mutations of *CHD8* Cause Autism and Macrocephaly

Whole exome sequencing (WES) [23,24,25,26] and targeted resequencing [6,7,10,25,27,28,29] of parent–child trios/quads have established *CHD8* as one of the most frequently mutated and most penetrant genes in ASD. Heterozygote de novo mutations included frameshift, nonsense, missense, translocation, single nucleic acid deletion, and splice site variant mutations; most of these were predicted to cause a loss of function. Mutations in *CHD8* were most frequently associated with autism, followed by macrocephaly [12,27,29], a phenotype also observed in patients carrying a balanced translocation disrupting *CHD8* [30,31]. Orbital overgrowth developed within the first 2 months postnatally, indicating a neurodevelopmental origin, and concurred with increased head growth throughout early childhood. Patients with *CHD8* mutations also represent facial dysmorphisms (prominent forehead and eyes and posteriorly rotated ears) and, to a lesser degree, recurrent obstipation and sleep disturbances. A body of recent studies further supports that disruptive de novo mutations of *CHD8* underpin a neurodevelopmental syndrome with the leading symptoms of autism and macrocephaly [32,33,34,35]. More specifically, Beighley et al. [33] showed that individuals with ASD carrying *CHD8* mutations display less severe adaptive deficits in communication skills and lower seizure prevalence when compared to individuals with ASD carrying other high-risk mutations. This finding suggests that a more nuanced picture of behavioral and cognitive phenotypes in individuals with *CHD8* mutations is important to guide future preclinical and basic research.

## 4. The Excitation/Inhibition Hypothesis in ASD

Rubenstein and Merzenich [36] originally hypothesized deregulation of the balance between excitation and inhibition (E/I balance) in cortical and subcortical circuits as a key mechanism in patients with ASD. Different homeostatic and developmental processes are known to maintain the E/I balance during development and beyond (reviewed in [37]). At the level of single neurons, information processing critically depends on the balance between excitatory and inhibitory inputs and is spatially regulated by various processes, including intrinsic neuronal excitability, synaptic transmission, and homeostatic plasticity ([38], reviewed in [39]). At the circuit level, the E/I balance reflects the complex interaction between glutamatergic excitatory and GABAergic (γ-aminobutyric acid) inhibitory neurons, excitatory and inhibitory synapse formation, and overall neuronal network activity [40,41]. In the following, we will consider three independent research lines that have provided complementary evidence for the E/I balance hypothesis in ASD.

First, functional brain studies support the E/I imbalance hypothesis in patients with ASD ([42]; reviewed in [43]). For example, γ-band electrophysiological activity (30–100 Hz), a presumed proxy to the E/I balance within local neuronal circuits, is altered in patients with ASD, particularly in relation to auditory-related γ-band activity [44,45]. In addition, magnetic resonance spectroscopy (MRS) has enabled the measurement in vivo of the abundance of the excitatory and inhibitory neurotransmitters glutamate and GABA, respectively. This more direct approach corroborated alterations in neurotransmitter levels within different cortical and subcortical regions in patients with ASD [46,47,48,49]. For example, glutamate levels in the striatum were reported to correlate negatively with social impairment [47], suggesting that the E/I balance hypothesis is of clinical relevance. In light of the significant heterogeneity in ASD, patient stratification is needed to achieve better granularity in functional phenotyping.

**Figure 3:**
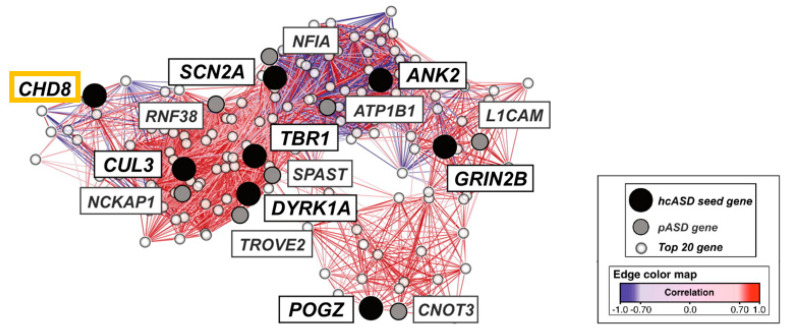
Mid fetal spatio-temporal coexpression network in the human prefrontal cortex. The analysis comprised weeks 13 to 19 post conception. High confidence ASD risk genes (*hcASD seed gene*) are marked in black with *CHD8* boxed in orange. Probable ASD risk genes (*pASD gene*) are shown in grey, and the top 20 genes (*Top 20 gene*) best correlated with each hcASD gene in white. The lines (edges) represent coexpression correlations ≥0.7; positive correlations are shown in red and negative correlations are shown in blue. Graphic adapted from [51], attribution license 5004710196206.

Second, integrated functional genomic analysis has been used to narrow down specific molecular pathways and circuits in ASD. Parikshak et al. [50] mapped ASD and ID risk genes onto coexpression networks representing developmental trajectories and transcriptional profiles derived from fetal and adult cortical laminae. Notably, multiple modules enriched in ASD risk were connected by this approach to mid fetal glutamatergic projection neurons in the upper (L2/L3) and lower (L5/L6) cortical layers. In a contemporary study, Willsey et al. [51] sought to identify periods, brain regions, and cell types in which nine high confidence ASD risk genes, including *CHD8*, converge. Coexpression networks inferred from these “seed” genes highlighted multiple brain regions, such as the cortex, across human development and into adulthood. Assessing enrichment of an independent set of probable ASD genes enabled narrowing down a key point of convergence in mid fetal deep (L5/L6) layer cortical glutamatergic projection neurons (Figure 3).

The differences in the assignment of the specific cortical layers between these two studies [50,51] are unsurprising given the differences in the input genes selected, in the approaches to network construction, and in the selection of expression data. Collectively, these findings implicate *CHD8* in glutamatergic projecting regions, which play an important role in social behavior and intellectual ability, among other higher brain functions.

Most recently, imaging genomics has highlighted genes that covary with resting-state functional MRI (rs-fMRI) measurements across cortical projecting regions (reviewed in [52]), indicating that gene expression might underpin functional signals in the human brain. To expand this approach to ASD, Berto et al. [53] integrated brain gene expression datasets of neurotypical controls and individuals with ASD and regionally matched brain activity measurements from fMRI datasets. This enabled the identification of genes linked with brain activity, whose association was disrupted in individuals with ASD. A subset of these genes showed a differential developmental trajectory in individuals with ASD relative to controls. These genes were enriched in voltage-gated ion channels and inhibitory neurons, supporting the E/I imbalance in ASD.

Third, independent support for the E/I imbalance hypothesis has grown from transgenic mice studies. Platt et al. [54] used CRISPR/Cas9 editing to produce a germline *Chd8* heterozygote frameshift mutation in mice. Notably, these animals showed impaired Wingless (Wnt) signaling in the nucleus accumbens (NAs), which, together with deregulated cell adhesion, led to a reduction in local inhibitory signaling of medium spiny neurons (MSNs). Consequently, MSNs displayed an increase in spontaneous excitatory output associated with mild deficits in social interaction, elevated anxiety, and increased motor learning. In a subsequent study, Jung et al. [55] produced germline *Chd8* heterozygote mice (Asn2373LysfsX2) recapitulating a mutation in patients with ASD (Asn2371LysfsX). Interestingly, hippocampal neuronal activity was suppressed in females under resting conditions and rose to neural activity of wild type mice following exposure to an environmental stressor. At the opposite, heterozygote male mice displayed normal resting neuronal activity but an enhanced response following stress exposure. Consistent with this finding, the synaptic inhibitory transmission was enhanced in female hippocampi but reduced in male hippocampi. Behavioral phenotyping of germline heterozygote *Chd8* mice showed male preponderant abnormalities of social communication in pups, anxiety-like mother seeking/attachment behavior in juveniles, and isolation-induced self-grooming in adults. Taken together, this study showed that germline *Chd8* heterozygosity associates with sex-preponderant behavioral deficits and sexually dimorphic inhibitory synaptic transmission in the hippocampus, a brain region strongly connected to different brain regions involved in ASD. In a most recent study, Ellingford et al. [56] investigated germline heterozygote *Chd8*-floxed mice that were mated to *Cre-*mice in which expression of *Cre* was directed to either all cell types (*β-actin-Cre^+/^*^−^) or specifically to glutamatergic (*NKx.1-Cre^+/^*^−^) or GABAergic (*NEX-Cre^+/^*^−^) neurons. Synaptic development of prefrontal pyramidal neurons was affected in a stage-specific and cell-autonomous manner in germline *Cdh8* heterozygote mice and caused contrasting changes in excitatory and inhibitory synaptic transmission. Unexpectedly, heterozygote neurons responded with increased, rather than decreased, inhibitory transmission to a blockade of spontaneous transmission in ex-vivo brain slices. This inhibitory response points to the presence of dysregulated mechanisms of homeostatic plasticity in germline *Chd8* heterozygote prefrontal neurons.

In summation, results from germline *Chd8* heterozygote mice strengthen the E/I balance hypothesis of ASD in general and point to sex-, stage-, tissue- and cell-type-specific abnormalities in synaptic transmission and homeostatic plasticity. While it is tempting to extrapolate from these findings to humans, more detailed insight into the molecular mechanisms driving the E/I imbalance in mice and human models of brain development are needed.

## 5. Single-Cell Sequencing of Brain Cells with *CHD8* Mutations

### 5.1. Defining the Molecular Identity of Neural Cells

Multipotent cells of any organ, including the human brain, develop and differentiate along specific lineage trajectories to produce distinct cell types whose well-function is critical to sustaining human health. Cellular mechanisms underpinning the onset and course of neurodevelopmental disorders are still poorly understood, thus limiting timely and more efficient therapies of ASD. Progress in this area will require an understanding of “when, why and how cells deviate from their normal trajectories” [57] (p. 377). To realize this ambitious goal, it will be necessary to establish a detailed molecular roadmap of cellular development across space and time with sufficient resolution. High-throughput single-cell sequencing technologies directly quantify information-bearing molecules, such as DNA and/or RNA, that encode and enact each cell’s biological identity. Fine-grained, broad-scope single-cell sequencing data can advance our understanding of the identity of hundreds of cellular phenotypes that form the building blocks of neural circuits (reviewed in [58]). Beyond that, single-cell sequencing of cells from in vivo and/or in vitro models of neurodevelopment can inform us “when, why and how” the presence of high confidence risk genes interferes with normal brain development in ASD.

### 5.2. Single-Cell Sequencing of CHD8 Knockout during Mouse Neocorticogenesis

The combination of single-cell RNA sequencing (sc-RNAseq) and CRISPR/Cas9 technologies, called Perturb-Seq, allows the production of information-rich transcriptomes and to introduce and analyze at the same time the effects of genetic perturbations. In CRISPR/Cas9, a guide RNA (gRNA) is designed to bind to the DNA sequence that is to be edited. The Cas9 enzyme then binds to the gRNA and induces a break in the DNA. The cell often incorrectly repairs this break and produces a gene “knockout” with a partial or entire loss of protein function. In Perturb-Seq, each cell is then sequenced independently so that different genetic manipulations and numerous genes can be investigated in one experiment across all cell types and states.

**Figure 4:**
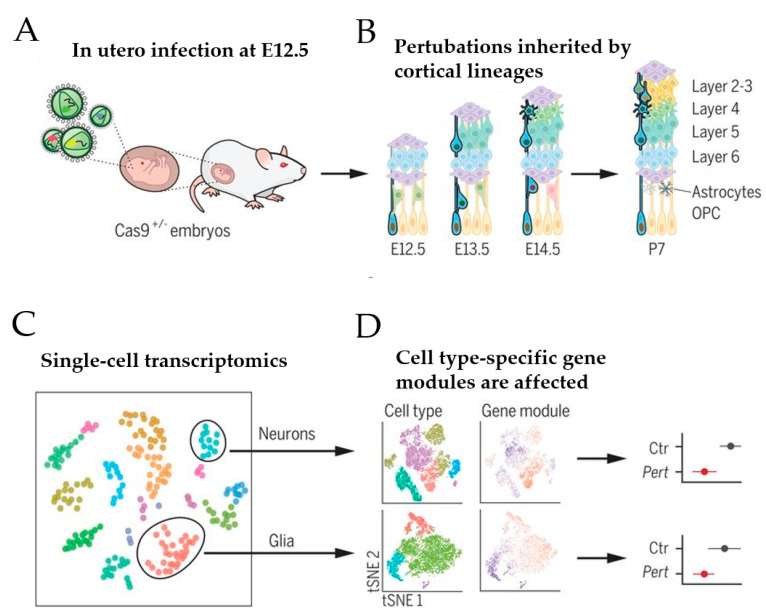
Schematic of in vivo Perturb-Seq analysis of mouse corticogenesis. (**A**) The lateral ventricles of Cas9^+/^^−^ embryos were infected at E12.5 with lentiviral particles that contained a lentiviral guide RNA (gRNA) library targeting ASD/NDD risk genes. (**B**) ASD/NDD risk genes were gene-edited (“knocked-out”) in infected neural progenitor cells. These mutations were passed on to their progeny, including cells of the cortical lineage that form the upper and lower layer of the cortex. (**C**) At postnatal day 7, cortices were dissected and used for single-cell sequencing. (**D**) Gene expression modules were affected in a manner dependent on the individual gene perturbation and the specific cell type. Scheme adapted from [59], attribution license 5004760017864.

Based on this approach, Jin et al. [59] recently sought to address the role of a panel of high-risk ASD/NDD genes, including *CHD8*, during moue corticogenesis in vivo (Figure 4)

A transgenic mouse line that constitutively expressed Cas9 was chosen to infect the developing lateral ventricles of Cas9 heterozygote embryos in utero with pools of gRNAs, each targeting a specific high-risk ASD/NDD gene (Figure 4A). To enhance knockout efficiency, each lentiviral vector harbored two different gRNAs complementary to the coding exons of one ASD/NDD gene and a blue fluorescent protein (BFP) reporter with a distinct barcode corresponding to the perturbation target. The lentiviral injection was carried out on embryonic day 12.5, leading to the infection of neural progenitors lining the lateral ventricle of the developing neocortex and the ganglionic eminence. This approach enabled investigation of the effect of each perturbation across a wide range of cell types from distinct brain regions, including cortical projection neurons, interneurons, astroglia, and oligodendrocytes (Figure 4B). Under sparse labeling conditions, less than 0.1% of cells in the cortex were infected, implicating that these cells developed amidst unperturbed neighboring cells. At postnatal day 7, infected cortical cells were isolated by fluorescence-activated cell sorting (FACS) and used in a droplet-based scRNA-seq (see [58] for an introduction to this technique) to assess each cell’s expression profile along with its perturbation code. This analysis revealed that 40 to 70% of the FACS-positive cells contained frameshift insertion or deletion for each gRNA target. After filtering out low-quality cells, 35,847 cells remained that comprised cortical projection neurons, cortical inhibitory neurons, astrocytes, oligodendrocytes, and microglia (Figure 4C). Among these cells, 50% carried barcodes for a single gene perturbation with a median of 338 cells per perturbation. Together, these perturbations embraced 35 ASD/NDD risk genes, including *Chd8*. Since the small number of cells for any given perturbation per cell type prevented differential expression analysis between gene-edited and normal cells, Jin et al. [59] calculated the effect size of each perturbation on correlated expression modules across cell types compared with cells infected with control vector (i.e., lentiviral vector expressing green fluorescence protein) (Figure 4D).

Interestingly, perturbation of *Chd8* affected the expression of a gene module highly expressed in oligodendrocyte precursor cells (OPCs) but lowly in committed oligodendrocyte progenitors (COPs) and newly formed oligodendrocytes (NFOLs). This finding suggested a role of *Chd8* in the development of the oligodendrocyte lineage, a hypothesis further supported by in-situ hybridization and immunohistochemistry analysis of marker genes (*Cspg4*, *Pdgfra*, and *MBP*) in cortices from *Chd8* germline heterozygote mice. In agreement with these findings, previous studies on *Chd8* germline heterozygote mice [60,61] have found that combinatorial interactions of *Chd8* with lineage-specific TFs (e.g., Olig2), chromatin modifiers (e.g., KMT2), and chromatin remodelers (e.g., CHD7) coordinate the temporal and spatial control of oligodendrocyte lineage-specific gene regulation. *Chd8* heterozygosity is associated with early proliferation defects of OPCs, impaired oligodendrocyte differentiation, and circumscribed myelination defects. Notably, cognitive, behavioral, and motor deficits in patients with ASD have been attributed to myelination defects, including frequent central white matter abnormalities consisting of deficits in myelin content and compaction [62,63]. Likewise, one-third of the individuals with a mutation in *CHD8* present variable degrees of ventriculomegaly and delayed myelination [64]. Taken together, these findings indicate that disruption of *Chd8* during mid fetal neocorticogenesis in mice elicits postnatal perturbations in gene expression modules relevant to clinical phenotypes.

Unexpectedly though, *Chd8* editing during mid fetal neocorticogenesis did not perturb gene modules related to excitatory and/or inhibitory neurons. This result is in stark contrast to those from previous studies on germline heterozygote *Chd8* mice (see Section 4 and [65]) and from integrated functional genomics of ASD/NDD risk genes [50,51]. We consider further possible explanations for these discrepancies in the discussion section.

### 5.3. Organoids as a Model for Human Brain Development

Embryonic stem cells (ESCs) and induced pluripotent stem cells (iPSCs) represent powerful tools to model in vitro early steps of human neurodevelopment. These cells can be differentiated in virtually any cell type relevant to ASD and have eased restraints from the limited availability of embryonic brain tissue and from postmortem brain tissue to inform on early disease processes. Furthermore, ESC/iPSC derived cells are not inflicted by secondary alterations owing to disease course, therapy, or patients’ life history and provide unlimited access to cell populations ranging from neural stem cells (NSCs) to neural progenitor cells (NPCs) to neurons (reviewed in [66]). However, neuronal differentiation in monolayer culture does not recapitulate the three-dimensional (3D) organization of the human brain and well-known structure–function relationships [67] that are important to understand the cellular origin of ASD. In this respect, pluripotent stem cell-derived brain organoids represent a tractable reductionist system that allows the modeling of molecular and physiological aspects in space and time [57].

Human brain organoids recapitulate in vitro many features of fetal brain development, including cytoarchitecture, cell diversity, and maturation (reviewed in [68]). In this respect, cerebral organoids show the progressive features of cortical development, starting with the formation of ventricular and subventricular zone-like structures and the development of organized neuronal cell layers. Cortical organoids at day 10 (D10) consist almost exclusively of Sox2-positive radial glial cells recapitulating the developing human cortical ventricular zone. These multipotent progenitors give rise to increasing proportions of intermediate progenitors (TBR2 positive) and lower layer (CTIP positive), and upper layer (SATB2 positive) cortical neurons.

Among mature neurons, glutamatergic neurons are the most abundant cell type, while GABAergic interneurons are few. Glial cells are at least equally abundant as neurons and contribute to neuronal activity, synaptogenesis, and circuit remodeling. OPCs have also been detected in cortical organoids, where they support partial myelination of axons. Mature oligodendrocytes develop only under long-term culture and then do not implement the complex organization of myelin sheets, which is necessary to enhance the propagation of nerve impulses.

A meta-analysis of single-cell transcriptomes supports that cerebral organoids recapitulate gene expression programs of in vivo neocorticogenesis up to 24 weeks after conception [69]. The long-term culture of cerebral organoids is compromised by increasing tissue heterogeneity [70] and necrosis in the organoid core [71]. In the absence of functional vascularization, insufficient oxygenation and nutrient diffusion remain severe bottlenecks that curtail later maturation stages. These bottlenecks might also contribute to high organoid-to-organoid variability that has raised concerns about the consistency of developmental processes outside the context of human embryogenesis [70]. On the other hand, Velasco et al. [72] recently showed that patterned forebrain organoids exhibit a nearly indistinguishable repertoire of cell types when compared to human brains and trace developmental trajectories with similar variability.

Overall, organoids represent a tractable entry point for studying the role of ASD risk genes in early brain development [68].

### 5.4. Bulk Sequencing of CHD8 Knockout Cerebral Organoids

In 2017, Wang et al. [73] first carried out gene expression profiling on tissue homogenate from *CHD8* heterozygote cerebral organoids. Although this approach did not apply sc-RNAseq, we nevertheless chose to include this work given its relevance to the E/I imbalance hypothesis. Wang et al. utilized a previously generated isogenic pair of iPSCs from a healthy male donor in which one copy of *CHD8* had been edited via CRISPR/Cas9 to produce an N-terminal truncation [74].

Bulk-sequencing of cerebral organoids aged 50 days revealed gene expression profiles resembling the first-trimester telencephalon. Among 559 differentially expressed genes (DEGs), 288 were up- and 271 were downregulated in *CHD8* heterozygote cerebral organoids, and 203 were predicted to contain a CHD8 bindings site in their promoter region. DEGs included *TCF4* (a basic helix-loop-helix TF), *POU3F2* (a member of the POU family of TFs), and *AUTS2* (a chromatin-remodeling factor that acts in the context of the Polycomb repressive complex 1 (reviewed in [75]); all three factors have also been implicated in schizophrenia and bipolar disorders. Analysis of enriched pathways and disease association suggested that *CHD8* directly or indirectly controlled critical aspects of brain development, including neurogenesis, neuronal differentiation, forebrain development, axonal guidance and wingless/β-catenin signaling.

Interestingly, two of the three top-ranked DEGs in *CHD8* heterozygote cerebral organoids were *DLX6-AS1* (distal-less homeobox antisense 1) and *DLX1* (distal less homeobox 1) that were upregulated ~39- and ~13-fold, respectively. Members of the DLX gene family contain a homeobox that is related to that of Distal-less (Dll), a gene expressed in the head and limbs of the developing fruit fly. Likewise, the six members of the mammalian *Dlx* gene family are expressed in the nervous system, neural crest derivatives, branchial arches, and developing appendages and have been implicated in patterning and development of the brain, craniofacial structures, and the axial and appendicular skeleton (reviewed in [76]). In mice, a splice variant of Dlx6-AS1, called Dlx6-AS2, cooperates with Dlx1 and Dlx2 proteins in coactivation of the Dlx5/Dlx6 enhancer [77] and promotes differentiation of GABAergic interneurons in the developing forebrain [78,79]. Consistent with this scenario, genes with a role in cerebral GABAergic interneuron differentiation, including *FEZF2*, *ARX*, and *CNTN2,* were differentially expressed in *CHD8* heterozygote organoids.

Independent support of the finding by Wang et al. [74] is provided by a prior study by Mariani et al. [80], who detected as well upregulation of *DLX6-AS1* among top-ranked DEGs. In this RNA-seq study, iPSCs were derived from four families, each of which included an individual with idiopathic ASD and increased head circumference. Importantly, GABAergic neurons were overproduced in cerebral organoids derived from individuals with idiopathic autism due to an increase in *FOXG1* gene expression, a key regulator of forebrain development.

Taken together, these two studies suggest that a shift toward GABAergic neuron fate is common to both individuals with *CHD8* mutations and idiopathic autism. Future studies are needed to uncover through which mechanisms CHD8 and FOXG1 upregulate DLX6-AS1 expression.

### 5.5. Single-Cell Sequencing of CHD8 Knockout Cerebral Organoids

Most recently, Villa et al. [81] carried out sc-RNAseq of human cerebral organoids to assess the effect of various *CHD8* mutations on human neocorticogenesis. CRISPR/Cas9 editing in a human ESC line was used to delete either the C-terminal helicase domain or introduce two patient-specific mutations, each resulting in a premature stop codon (i.e., *S62X* and *E1114X;* see Figure 2). Notably, both mutations are associated with autism; yet, only the E1114X mutation is associated additionally with macrocephaly and ID [12,35], suggesting differences in etiopathogenesis.

Addressing previous concerns on organoid-to-organoid variability, the researchers first optimized culture conditions so that more than 90% of the embryo bodies successfully differentiated into cerebral organoids. At D20, *CHD8* mutant organoids were either equally large or slightly smaller than their wild type counterparts. Thereafter, *CHD8* mutant organoids, except those containing the S62X mutation, gained in size and surpassed wild type organoids by ~50% at D120. This differential growth pattern suggested that patient mutations associated with macrocephaly drove organoid overgrowth, while the patient mutation unassociated with macrocephaly preserved normal organoid growth.

Droplet-based scRNAseq of three different developmental stages (i.e., D20, D60, and D120) evidenced 10 different cell populations, including three clusters of radial glial cells, intermediate progenitors, interneuron progenitors (IN-IPs), interneurons (INs), early excitatory neurons, and excitatory neurons of upper (EN1) and lower (EN2) layers. Interestingly, subsequent analysis of the relative densities of these cell populations in wild type and oversized *CHD8* heterozygote organoids showed stage-specific alterations, including an advanced production of IN and IN-IP and a delayed production of EN1-EN2. Consistent with this finding, temporal analysis of developmental branches supported an overrepresentation of *CHD8* heterozygote cells in the interneuron branch at D60 and in the excitatory branch at D120. Among changes in cell populations due to *CHD8* heterozygosity, the increase in interneurons of the parvalbumin lineage at D60 represented the most robust shift.

Further immunohistochemistry and cell proliferation studies suggested that this shift in cell populations originated from a proliferative imbalance of neural progenitors that led to an expansion of this compartment and in the number of later neurons. Concurrently though, the number of Tbr2-positive intermediate progenitor decreased and led to a reduced thickness of later cortical layers. This differential shift occurred in a cell-autonomous manner as evidenced from mosaic organoids containing wild type and *CHD8* heterozygote cells in equal parts.

Bulk sequencing identified 868 DEGs at D10, among which equal amounts were up- or downregulated in *CHD8* heterozygote organoids and were significantly enriched in high confidence ASD genes, such as the chromatin regulator *ASHL1*. In agreement with previous experimental studies on *CHD8* loss of function mutations (reviewed in [14,65]), upregulated genes were enriched for those involved in cell cycle progression, RNA splicing, and transcription, while downregulated genes were enriched for those involved in neuronal differentiation and brain development. Among upregulated genes, 47% were predicted to be CHD8-bound, suggesting direct regulation, whereas, among the downregulated genes, only 28% were predicted to be CH8-bound, suggesting indirect regulation.

Moreover, stage-specific sc-RNAseq analysis corroborated the division between proliferation and neurogenesis enrichments from bulk sequencing and showed that up- and downregulated genes mapped onto DEGs in different D20 cell clusters. Interestingly, upregulated genes related to cell cycle, mRNA metabolism, ribosome biogenesis, and translation initiation were enriched in clusters of radial glial cells, whereas downregulated were enriched in clusters from the differentiation path. For example, ZEB2 (zinc finger E-box binding homeobox2) was strongly downregulated in *CHD8* heterozygote radial glial cell. ZEB2 encodes a DNA-binding transcriptional repressor that interacts with activated SMADs, the transducers of TGF-β signaling, and with the nucleosome remodeling and histone deacetylation (NURD) complex (for the role of NURD in brain development see [22]). In particular, ZEB2 promotes the neuroepithelial differentiation into radial glial and thus presets the number of progenitors that participate in cortical expansion [82].

In summation, *CHD8* heterozygote NPCs from cerebral organoids showed an imbalance between proliferation and differentiation characterized by (i) an expansion of the NPC pool with dysregulation of neuronal differentiation and cell cycle pathways in radial glial cells, (ii) a protracted proliferation of excitatory neuron progenitors leading to a reduced formation of cortical neurons from both upper and lower layers, and (iii) a robust increase in interneuron production, particularly of the parvalbumin GABAergic lineage. These deviations developed in a cell-autonomous manner, suggesting that only selected cell populations during specific developmental stages were vulnerable to *CHD8* dosage.

## 6. Discussion and Outlook

The advent of sc-RNAseq, CRISPR/Cas9 editing, and human organoids provides unprecedented opportunities to determine both in vivo and in vitro the effects of *CHD8* mutations on single cell types during the early stages of brain development. Previous clinical and transgenic mice studies have suggested that alterations in the E/I balance are relevant to ASD, including patients with *CHD8* mutations. Most recent sc-RNAseq studies focusing on mice neocorticogenesis and human cerebral organoids have drawn a nuanced picture of *CHD8′*s role in neurodevelopment. In the following, we will discuss potential pitfalls, pros and cons, and future improvements to harvest the full potential of these promising approaches.

Jin et al. [59] introduced in vivo Perturb-Seq as a highly scalable method to assess the role of high confidence risk genes in ASD/NDD during mouse neocorticogenesis. In utero, editing of *Chd8* turned out to disrupt oligodendrocyte gene expression modules reminiscent of findings from heterozygote *Chd8* mice [60,61]. Unexpectedly though, *Chd8* editing did not affect the expression of genes related to the excitatory/inhibitor system. Several caveats come to mind that might explain this perplexing result.

While unlikely, we cannot rule out the possibility that *Chd8* editing at E12.5 leaves out earlier periods of neural development that could be particularly vulnerable to *Chd8* dosage and thus influence the development of the excitatory/inhibitory system. In fact, previous mice studies have reported substantial differences in NPC proliferation and behavior between in utero knockdown of Chd8 at E13 and heterozygote *Chd8* germline mutations (reviewed in [65]). This discrepancy might reflect differences in the developmental stage at which *Chd8* was inactivated and to what degree.

Another concern relates to the *Chd8* genotype in FACS-sorted neocortical cells. Jin et al. reported a 40 to 70% frameshift insertion or deletion for each gRNA target among neocortical cells, whereby *Chd8* editing varied between 50–100% in two experiments. However, we do not know whether editing resulted in heterozygote or homozygote inactivation of *Chd8* and if this happened in a cell-type-specific manner. The precise nature of *Chd8* mutations is thought to play an important role with respect to cellular and organismal phenotypes. Hurley et al. [83] reasoned that *CHD8* mutations may encode loss-of function (haploinsufficient), hypomorphic, or dominant negative effects on protein function. Analysis of a series of *Chd8* alleles in mice, including *Chd8* heterozygote, mild or severe *Chd8* hypomorphs, and *Chd8* null alleles, showed that brain development was highly sensitive to *Chd8* dosage. Thereby, varying sensitivities of different progenitor populations are associated with non-linear effects on gene transcription and brain growth [83]. Uncertainty about *Chd8* genotypes and varying sensitivity to *Chd8* gene dosage in different cell populations could explain, at least in part, why in vivo Perturb-Seq evidenced a role for *Chd8* in the development of the oligodendrocyte lineage, but not of the excitatory/inhibitory system.

In this context, it is also important to recall that the small number of cells for any given perturbation per cell type did not allow a simple differential expression analysis between gene-edited and normal cells to be carried out. Instead, the perturbation effect size was calculated on correlated expression modules across cell types compared with cells receiving control vector [59]. Low cell numbers for certain cell types and potential differences in *Chd8* dosage make it difficult to assess perturbation-associated phenotypes properly.

In addition to the above issues, a principal reservation needs to be considered too. Sparse labeling of developing neocortical cells implicated that infected cells were neighbored almost exclusively by uninfected cells. While this approach favored the identification of cell-autonomous transcriptional effects from gene-edited ASD/NDD risk genes, it might mask more complex phenotypes that arise from reciprocal cell–cell interactions. The developing and mature brain depends on an intricate balance of interactions between neuronal, glial, immune, and vascular cells that underpin mental health. In this respect, it is important to note that the E/I imbalance hypothesis of ASD [36,84] does not posit a cell-autonomous origin. Transcriptomics studies on ASD-associated coexpression patterns in postmortem brain have identified networks of brain development genes [85] implicated in ASD and specified mid fetal development as a critical period for initiation of ASD neuropathology [50,51]. In addition, these studies have identified networks of genes related to immune response [85,86] and activation of M2 microglia [85] to be differentially coactivated in ASD brains. Although questions remain as to whether this has etiologic implications or is a downstream consequence of other events, these findings suggest a critical role of cell–cell interactions in the development of ASD.

In a nutshell, unresolved questions of vulnerable time windows, *Chd8* gene dosage, *Chd8*-dependent cell–cell interactions, and statistical power could confound in vivo Perturb-Seq experiments and thus explain the lack of evidence for a role of *Chd8* in the developing neocortical excitatory/inhibitory system.

How do then cerebral organoids compare to in vivo Perturb-Seq for the study of *CHD8*-associated phenotypes? Clearly, cerebral organoids are not an in vivo system but a broadly-accepted reductionist model of early brain development when the effects of *CHD8* mutations are laid out. Until recently, the production of cerebral organoids has been labor-intensive and plagued by high organoid-to-organoid variability. Refinements in culturing techniques [81] and progress toward fully automated high-throughput workflows for human organoid production and analysis [87] open the perspective to enlarge sample sizes in terms of the number of independent donors and genes to be investigated. Improvements in reproducibility are likewise important to cost reduction and statistical analysis [88].

An obvious advantage of ESC/iPSC-derived cerebral organoids is that they recapitulate a significant part of human brain development that might be important to ASD and patients with *CHD8* mutations. Although rodent models have a long-standing history as valid systems for disease modeling, the domestic mouse, the workhorse among animal models, shows some substantial differences with humans in terms of brain development. At the molecular level, gene expression profiles diverge between human and mouse for genes involved in cortical development and function [89,90,91] and might confound the analysis of neurodevelopmental dynamics relevant to disease. Moreover, at the cellular layer, the mouse brain contains just ~14 million cortical neurons in comparison to ~12 billion in humans and is lissencephalic, while that of humans is gyrencephalic [68]. Radial glial cells represent the first neural progenitor population in both species; yet, outer radial glial progenitors (oRGs) localized in the outer subventricular zone (oSVZ) are significantly more abundant in primates relative to rodents. Terminal differentiation into neurons is greatly expanded in humans relative to rodents and contributes to the increase in the cortical surface and the over-representation. Interestingly, Hurley et al. [83] reported an increased progenitor proliferation in *Chd8* hypomorph mice that was primarily confined to Tbr2- positive progenitors. In light of the importance of this compartment for human cortical growth, the authors suggested that human brain development might be more vulnerable to *CHD8* dosage than the mouse. Strikingly, Villa et al. [81] described in human cerebral organoids a shift in the proliferation/differentiation dynamics of neural progenitors that drove the expansion of this compartment and a subsequent increase in the number of later neurons. At the same time, however, the number of Tbr2-positive intermediate progenitors actually decreased. Moreover, by adopting an isogenic design of patient-specific mutations for the generation of mosaic organoids, the researchers showed that heterozygote *CHD8* mutations led to a cell-autonomous sustained proliferation of neural precursors in human cerebral organoids. At this step, it would be interesting to know to what degree mosaic organoids exactly recapitulate the dynamics of the proliferation/differentiation imbalance that led to an accelerated production of inhibitory neurons and a delayed production of excitatory neurons. While further experiments are necessary to reconcile the differences between transgenic mice and human organoids, as well as to define the molecular mechanisms underpinning the developmental imbalance in *CHD8* heterozygote cerebral organoids (e.g., through rescue experiments), the current data situation strengthens the case for cerebral organoids to uncover pathogenic mechanisms specific to men.

Notwithstanding recent progress on cerebral organoids, it is important to keep in mind that in vitro cultured cells can only approximate the cellular identity and complexity of the living brain. While further improvements on cerebral organoids and sc-RNAseq will empower transcriptomic analysis, we would like to emphasize that ASD risk genes do not per se encode psychopathology. ASD risk genes are numerous, with each variant conferring to a different degree changes at the molecular and cellular level that converge into the formation of micro- and macro-circuits during early brain development and beyond. Behavior emerges from neuronal circuits processing information from the environment, and aberrant synapse and circuit physiology are thought to lie at the heart of psychopathology. Undoubtedly, access to behavioral phenotypes is one of the key strengths of *Chd8* heterozygote mice. Contrariwise, cerebral organoid studies on high confidence risk genes, such as *CHD8,* still face the challenge of demonstrating that molecular and cellular differences detected in vitro are relevant to disease-related behavioral changes in vivo.

Electrophysiological studies on single cells and networks of cerebral organoids could contribute to close the gap between genes and behavior, at least in part. The developing cortex shows waves of spontaneous electrical activity in neurons and neural precursors that precede synapse formation [92]. With the onset of neuronal maturation and the projection of long-distance neurites, the spontaneous activity becomes synchronized across distance. Synchronized population bursts in mature whole-brain organoids [70] indicate that nerve impulses travel across interconnected neurons, and the lag time in signal transmission suggests that communication occurs through chemical synapses. As cortical organoids mature, they develop network-like activity, including highly synchronized oscillations and cross-frequency coupling. This indicates that activities at different frequencies interact and that networks communicate with each other [93]. Hence, future electrophysiological studies on isogenic *CHD8* heterozygote cerebral organoids shape up well to bridge proliferation/differentiation dynamics to intermediate phenotypes, such as E/I imbalance, underpinning higher-level systems function. Along this line, the combination of optogenetics and brain organoids could boost studies on neural network dynamics, functional communication between different organoid regions, and synaptic plasticity (reviewed in [94]).

Future translational research will also benefit from the transplantation of isogenic pairs of *CHD8* heterozygote cerebral organoids into neonate or adult mice brains. This experimental design allows the properties of heterozygote cells under more permissive conditions thought to unmask defects in migration, connectivity, circuit integration, to be studied and to promote later periods of advanced morphological and electrophysiological maturation. Mansour et al. [95] demonstrated that engrafted brain organoids developed corticothalamic and sub cerebral projection neurons followed by later intracortical projection neurons. Vascularization of human organoids took place within two weeks by the host tissue and supported human astrocyte and oligodendrocyte development together with host microglia invasion. This scenario raises the possibility of studying complex cell–cell interactions in vivo, moving beyond the current limitations of in vitro brain organoids. Relatedly, human iPSC-derived neurons were shown to form upon transplantation in mice brain long-range connections that underwent substantial structural maturation refinements pointing to host-derived pruning mechanisms [96]. In any case, molecular tagging of transplanted cerebral organoids/neurons will allow in vivo transcriptomics of single human cells under fairly physiological conditions in mice to be carried out.

The validity of transplantation experiments in mice as a surrogate measurement for behavioral deficits in humans has been illustrated by Windrem et al. [97]. Briefly, iPSCs derived from controls and patients with childhood-onset of schizophrenia (COS) were differentiated into human glial precursor cells (hGPCs) that were engrafted into neonatal immunodeficient shiverer mice (mice with congenital hypomyelination due to the absence of myelin basic protein). Control transplanted hGPCs developed into both astrocytes and myelinogenic oligodendrocytes and formed largely humanized forebrain white matter. Contrariwise, hGPCs derived from patients produced fewer precursors, were impaired in oligodendrocyte differentiation and showed little central myelogenesis. Astrocytes showed fewer primary processes, proximal branching and coherent domain structures, deficits that might affect their critical role in synaptic development and function [98]. Behaviorally, glial chimerization with patient cells significantly diminished auditory prepulse inhibition, a proxy to sensorimotor gating, and associated with increased anxiety and fear, deficits in socialization, cognition and sleep patterning. All of these symptoms are frequently found in patients with COS and suggest that defects in myelogenesis and astrocytic maturation impact higher systems level function (for further discussion, see [99,100]). Intriguingly, studies on *Cdh8* heterozygote mice [60,61] and *Chd8* editing during mice neocorticogenesis [59] suggest impairments in oligodendrocyte development whose implication for behavior could be likewise analyzed through engraftment of shiverer mice.

All in all, transgenic mice and pluripotent stem cell-derived models have provided a wealth of information on the molecular and cellular alterations from *CHD8* mutations. Beyond that, single-cell transcriptomics represents a powerful tool to further define at the molecular scale time windows and cell types particularly vulnerable to *CHD8* dosage. At the same time, future research needs to closely integrate these findings with those from patient stratification, deep phenotyping, and pathophysiological concepts in ASD and individuals with *CHD8* mutations. The E/I balance hypothesis illustrates par excellence present achievements, continuing challenges, and future opportunities along this way. Narrowing the gap between molecules and behavior is a step of great importance not only for developing a comprehensive picture of ASD but also for rational drug design, and more efficient and timely therapies.

## Data Availability

Not applicable.
